# CD36/Lyn kinase interactions within macrophages promotes pulmonary fibrosis in response to oxidized phospholipid

**DOI:** 10.1186/s12931-023-02629-6

**Published:** 2023-12-14

**Authors:** Doyun Kwak, Patrick B. Bradley, Natalia Subbotina, Song Ling, Seagal Teitz-Tennenbaum, John J. Osterholzer, Thomas H. Sisson, Kevin K. Kim

**Affiliations:** 1 Division of Pulmonary and Critical Care Medicine, Department of Internal Medicine, University of Michigan, 109 Zina Pitcher Place, BSRB 4061, Ann Arbor, MI 48109 USA; 2 Pulmonary Section, Department of Medicine, VA Ann Arbor Health System, Ann Arbor, MI 48105 USA

**Keywords:** CD36, Oxidized phospholipid, Pulmonary fibrosis, Macrophage, TGFβ, Lyn kinase

## Abstract

Recent data from human studies and animal models have established roles for type II alveolar epithelial cell (AEC2) injury/apoptosis and monocyte/macrophage accumulation and activation in progressive lung fibrosis. Although the link between these processes is not well defined, we have previously shown that CD36-mediated uptake of apoptotic AEC2s by lung macrophages is sufficient to drive fibrosis. Importantly, apoptotic AEC2s are rich in oxidized phospholipids (oxPL), and amongst its multiple functions, CD36 serves as a scavenger receptor for oxPL. Recent studies have established a role for oxPLs in alveolar scarring, and we hypothesized that uptake and accrual of oxPL by CD36 would cause a macrophage phenotypic change that promotes fibrosis. To test this hypothesis, we treated wild-type and CD36-null mice with the oxPL derivative oxidized phosphocholine (POVPC) and found that CD36-null mice were protected from oxPL-induced scarring. Compared to WT mice, fewer macrophages accumulated in the lungs of CD36-null animals, and the macrophages exhibited a decreased accumulation of intracellular oxidized lipid. Importantly, the attenuated accrual of oxPL in CD36-null macrophages was associated with diminished expression of the profibrotic mediator, TGFβ. Finally, the pathway linking oxPL uptake and TGFβ expression was found to require CD36-mediated activation of Lyn kinase. Together, these observations elucidate a causal pathway that connects AEC2 injury with lung macrophage activation via CD36-mediated uptake of oxPL and suggest several potential therapeutic targets.

**Supplementary Information:**

The online version contains supplementary material available at 10.1186/s12931-023-02629-6.

## Introduction

Progressive pulmonary fibrosis is a devastating condition that can occur in the setting of environmental exposures, in association with systemic inflammatory diseases, or as a primary process as with Idiopathic Pulmonary Fibrosis [1–3]. Although, the pathogenesis of fibrosis remains incompletely understood, recent evidence supports a multicellular mechanism involving the injury and apoptosis of epithelial cells, the recruitment and activation of profibrotic monocytes/macrophages, and the proliferation, differentiation, and survival of fibroblasts [4, 5]. Within these key cellular contributors to lung fibrosis, in vitro and in vivo studies have implicated a multitude of unique molecules and signaling pathways. However, despite a multitude of potential therapeutic targets, only a few treatments have established clinical benefit and are available to patients. The identification of new treatment options for these disorders will therefore require more precise and rigorous delineation of critical molecular and cellular pathways.

One molecule that holds promise as a therapeutic target in pulmonary fibrosis is CD36 as several complimentary animal models of pulmonary fibrosis reveal that targeted deletion of the CD36 gene leads to protection against scarring [6, 7]. Although the mechanism by which it regulates lung fibrosis remains poorly defined, CD36 possesses multiple functions through which it could potentially mediate scarring. For example, CD36 affects signal transduction pathways through its interaction with several cell surface proteins including integrins, tetraspanins, and toll-like receptors [8, 9]. Furthermore, CD36 has a binding domain for thrombospondin, a protein that has been implicated in the activation of the potent pro-fibrotic growth factor TGFβ [10]. Finally, CD36 functions as a scavenger receptor critical for the binding and clearance of apoptotic bodies (i.e. efferocytosis) and long chain fatty acids including oxidized phospholipids. As an example, CD36-mediated uptake of oxidized LDL by macrophages leads to foam cell formation, and this phenotypic alteration has been implicated in the pathogenesis of atherosclerotic lesions [11, 12]. In the context of vascular disease, the process of lipid uptake leading to the generation of foam cells requires the activation of intracellular tyrosine kinase signaling mediators including the src kinase family member, Lyn kinase [13, 14].

The scavenging receptor function of CD36 for apoptotic bodies has been implicated in the pathogenesis of pulmonary fibrosis. Specifically, Parks and colleagues demonstrated that CD36-null mice exhibited an attenuated capacity to clear apoptotic alveolar cells after bleomycin injury and that this decreased uptake of apoptotic bodies attenuated the severity of lung fibrosis [6]. Furthermore, we have recently shown that the installation of apoptotic AEC2 cells into the lungs of uninjured mice is sufficient to induce fibrosis, and the severity of scarring is attenuated in animals that lack CD36 expression [15]. How the uptake of apoptotic bodies by CD36 promotes scarring is unclear, but notably, apoptotic AEC2s are particularly phospholipid-rich as a byproduct of their critical function in surfactant lipoprotein production. AEC2 injury, which is a hallmark of lung fibrosis, can thereby lead to the release of excess phospholipid within the alveolar compartment [16]. Alveolar injury is also associated with oxidative stress, and the abundant phospholipid is susceptible to oxidation. Importantly, a recent report demonstrated that the instillation of oxPL into the lungs of uninjured mice was sufficient to cause fibrosis by day 14, implicating oxPL as a pro-fibrotic mediator [16].

Based on the role of CD36 in oxPL binding and clearance and the recent findings that oxPL can drive lung fibrosis, we hypothesized that CD36 is critical in mediating fibrogenesis through its role in scavenging oxPL. To test this hypothesis, we directly administered the oxPL species, oxidized phosphocholine (POVPC), into lungs of healthy adult WT and CD36-null mice. Consistent with our hypothesis, we found that oxPL induced fibrosis in a CD36-dependent fashion. Based on the known role of CD36 in macrophage foam cell formation in the context of atherosclerosis, we next investigated the effects of oxPL on CD36-mediated lung macrophage accumulation and phenotype. CD36 was determined to play a key role in increasing the lung macrophage population following oxPL administration, and these cells exhibit lipid uptake and an altered profibrotic phenotype. Like the foam cells found within atherosclerotic lesions, our studies showed that signaling through Lyn kinase, a src family tyrosine kinase, is a critical regulator of CD36-mediated oxPL scavenging and the acquisition of profibrotic activity.

In conclusion, our findings contribute to a growing literature that highlights CD36 as a key molecular driver of lung fibrosis. We also provide mechanistic evidence that links oxPL to the accumulation and activation of pro-fibrotic lung macrophage populations. Although additional studies are required to determine how the binding, uptake, and accumulation of oxPL by lung macrophages via CD36 leads to changes in phenotypic behavior, our results show that src kinase signaling contributes to the observed changes. Ultimately, our results implicate several targets in the oxPL-CD36 pathway that could be leveraged for therapy to potentially mitigate fibrosis.

## Methods and materials

### Mice

Mice were housed in a specific pathogen-free environment until the day of sacrifice. All in vivo experiments were approved by the University of Michigan Animal Care and Use Committee. Wild-type and CD36-null mice in a C57Bl/6 background were purchased from Jackson Laboratories and bred in our animal facilities. 1-palmitoyl-2-(5′-oxo-valeroyl)-sn-glycero-3-phosphocholine (POVPC), a form of oxPL, was delivered to mice by an oropharyngeal route at a dose of 10 µg/gram as previously described [17]. For bronchoalveolar lavage collection, mice were sacrificed at the timepoints indicated and lungs lavaged with 1 ml of PBS. Concentration of albumin in bronchoalveolar lavage fluid was determined using a Bromocresol Green Albumin Assay Kit (Sigma) following manufacturer’s protocol. Total cell counts were measured by hemocytometer as previously described [18, 19].

### Reagents

Nile Red Staining kit was from Abcam. Human/Mouse uncoated TGFβ ELISA kit was purchased from Invitrogen. Low melting temperature agarose is from Life Technologies. POVPC was purchased from Avanti Polar Lipids. Antibodies and sources are listed in Table 1. Bodipy 493/503 is from Cayman Chemicals. ProLong Gold anti-fade mounting media is from Invitrogn. TUNEL staining was performed with the TMR red In situ cell death detection kit from Roche. Protein A and protein G conjugated agarose beads are from Roche. Taqman Array for Mouse Lipid Regulated Genes was from Applied Biosystems. Sulfosuccinimidyl Oleate (SSO) is from Cayman Chemicals. Bafetinib is from Selleckchem. All other reagents are from Sigma Pharmaceuticals.

**Table 1:** Antibodies used for staining in flow cytometric analysis and immunoblot

Target	Clone	Fluorochrome	Manufacturer
CD45 (flow cytometry)	30-F11	PerCP-Cy5.5	BioLegend
Ly6G	1A8	APC	BioLegend
CD11b	M1/70	APC-Cy7	BioLegend
CD11c	N418	Brilliant Violet 421	BioLegend
Siglec F	E50-2440	PE-CF594	BD Biosciences
CD24	M1/69	Brilliant Violet 650	BD Biosciences
CD103	2E7	PE	BioLegend
MHC II (I-A/I-E)	M5/114.15.2	PE-Cy5	BioLegend
Ly6C	HK1.4	Alexa Fluor 700	BioLegend
CD206	C068C2	PE-Cy7	BioLegend
Arginase	polyclonal	PE	R&D Systems
Lyn	C13F9	none	Cell Signaling
Phospho-Lyn (Tyr397)	MA5-35,882	none	Thermo Fisher
CD36 (immunoblot)	EPR6573	none	Abcam
CD36 (immunofluorescence)	JJ2005	none	invitrogen
CD45 (immunofluorescence)	30-F11	BV421	BD Biosciences
Anti-rabbit IgG		Alexa Fluor® 647	Cell signaling

### SP-D assay

Levels of SPD within serum were measured using the Mouse SP-D Quantikine ELISA Kit (R&D Systems) according to the manufacturer’s instructions.

### Hydroxyproline assay

The collagen content of lungs was determined by an assay for hydroxyproline as previously described [18–22]. Briefly, lungs were removed from mice sacrificed at the timpoints indicated. Lungs were homogenized and incubated in 12 N HCl at 120ºC overnight. Samples were then neutralized with citrate buffer and incubated with chloramine T solution for 20 min at room temperature. Erlich’s solution was then added and the samples were incubated in a 65 °C water bath for 15 min. The absorbance at 540 nm was measured and the hydroxyproline concentration was calculated against a curve of standard concentrations of hydroxyproline.

### Nile red staining

Intracellular lipids were quantified with Nile Red Staining Kit from Abcam (ab228553) according to the manufacturer’s protocol for cells in suspension with minor modifications. Brefiely, lavaged BAL cells were centrifuged at 1100 rpm for 6 min and the cell pelletes were resuspended with 1 ml of RBC lysis buffer. Equal numbers of resuspended cells (5 × 10^4^) from each mouse were transferred into a 96 well plate with conically-shaped wells, centifuged at 1100 rpm for 6 min, and resuspended with 100 ul of Nile Red Staining Solution. Plates were incubated for 20 min at room temperature in the dark, centrifuged to remove the solution and resuspended with 100ul PBS at room temperature. The resuspended cells were transferred into black/clear bottom 96-well plate and spun down at 1000 rpm for one minute to collect the cells at the bottom of each well. Nile Red Stained cells were measured by reading with Spectra Max M3 plate reader.

### Cell isolation and culture

Primary murine bone marrow derive macrophages (BMM) were isolated and cultured as previously described [23, 24]. Briefly, the marrow was eluted from mouse femurs and dispersed into single cell suspensions. The cells were differentiated in DMEM containing 20% FBS and 30% L929 conditioned media. After 6 days, the media was replaced with fresh media and the cells were treated in the conditions indicated.

### TGFβ ELISA

For in vitro experiments, BMM from WT and CD36-null mice cultured in six well plates were treated with 100 uM sulfo-N-succinimidyl oleate (SSO), Bafetinib (10 µM) or vehicle control. After 1 h, cells were treated with oxPL (POVPC at 10 µg/ml) versus DMSO vehicle control. After 24 h, 100 µl of conditioned media was used to determine the concentration of TGFβ. For BAL fluid, 1 ml of PBS was instilled intratracheally into the lungs, aspirated, and centrifuged at 1500 rpm for 10 min to remove cells. The concentration of TGFβ was measured in cell-free BAL fluid or conditioned media samples by using the Human/Mouse TGF-β1 Uncoated Elisa kit (Invitrogen) per manufacturer’s instructions.

### Active caspase 3/7 assay

Caspase 3/7 activity was determined from lung lysate [25]. Briefly, at the timepoints indicated, lungs were isolated from mice and immediately frozen in liquid nitrogen and stored at − 80 °C. Lung were ground while frozen using a cryogrinder and then immediately lysed. Levels of active caspase 3/7 was determined by the Caspase Glo 3/7 assay (Roche) per manufactureres protocol and the luminescences quantified using a Veritas Microplate Luminometer and values normalized to protein concentration.

### Lung histology

The protocol for mouse lung histology was previously described [21, 26]. Briefly, mice were sacrificed and the trachea and thoracic cavity were exposed. The trachea was cannulated and the lungs were inflated to 25 cm H_2_O pressure with 4% paraformaldehyde. The lung were removed and were fixed in the 4% paraformaldehyde at room temperature overnight. The lungs were then embedded in paraffin, sectioned and stained with Masson’s trichrome by the McClinchey Histology Lab (Stockbridge, MI). Lung sections were visualized on a Nikon E-800 microcope and images captured with NIS Elements software.

### Cell staining and flow cytometry analysis

Fluorochrome-conjugated antibodies used for cell staining are listed in Table 1. Cells were first stained with a fixable viability dye (Zombie aqua; BioLegend, San Diego, CA) following the manufacturer’s protocol. After blocking Fc receptors using anti-CD16/32 antibody (clone 93, BioLegend), cells were stained for cell surface markers and then fixed with 2% formaldehyde (ThermoFisher Scientific) in PBS. Data were acquired using an LSRFortessa flow cytometer (BD Biosciences) and analyzed using FlowJo software (Treestar, Ashland, Oregon). At least 100,000 events in the CD45+ gate were acquired per lung sample. Gating of lung myeloid cells was performed as previously described [27] and further represented in Additional file 1: Figure S2. To determine the number of cells in each population of interest in each sample, the corresponding percentage was multiplied by the total number of viable CD45+ cells in that sample. The latter value was calculated for each sample as the product of the percentage of viable CD45+ cells and the original hemocytometer count of total viable cells identified within that sample. For Bodipy staining, BAL cells were collected and incubated with Bodipy (5 µg/ml) at 37 degrees for 20 min and then rinsed three times with PBS and analyzed by flow cytometry.

### Lung section immunostaining

Lung sections were stained and visualized as previously described [19]. Briefly, lungs were filled with OCT, removed and frozen in OCT. 12 µm lung sections were fixed with 4% PFA and permeabilized with 0.5% triton. Sections were blocked in buffer containing 5% normal goat serum and 1% BSA for at least 1 h at room temperature. Sections were then incubated with primary antibody diluted 1:100 in blocking buffer at 4 degrees overnight. Sections were rinsed three times with PBS and once with blocking buffer and then incubated in fluorescent-conjugated secondary antibody for at least 1 h at room temperature. In some cases, lung sections were analyzed by TUNEL staining using the In Situ cell death detection kit, TMR red per manufacturer’s protocol as previously described [19]. Lung sections were then washed with PBS and mounted with ProLong Gold with or without DAPI as indicated. In some cases, lung sections were co-stained with Bodipy (5 µg/ml) which was added to both the primary and secondary antibody solutions.

### Immunoblot and immunoprecipitation

Cells or lung tissue were analyzed by immunoblot and immunoprecipitation as previously described [20, 28]. Briefly, cells and tissue were lysed in RIPA buffer supplemented with sodium orthovanadate, PMSF, NaF and protease inhibitor cocktail. Debris was removed by centrifugation. For immunoblot, equal amount of protein from each lysate was loaded and separated by SDS-PAGE and transferred to nitrocellulose membranes. Membranes were blocked with buffer containing 5% milk and then incubated at 4 degrees overnight in primary antibody (1:1000 dilution) in blocking buffer. Membranes were rinsed three times and then incubated in HRP-conjugated secondary antibody (1:5000 dilution) in blocking buffer for at least 1 h at room temperature. Membranes were rinsed and developed with ECL solution and analyzed on an Amersham Imager 600. For immunoprecipitation, the lysate was precleared with protein A and protein G coated agarose beads. After beads were removed the precleared lysates were incubated with the primary antibody (or IgG control) at 4 degrees for at least 2 h with gentle rotation. The lysates were then incubated with protein A and protein G agarose beads overnight at 4 degrees. The beads were washed three times and analyzed by immunoblot as above. Densitometry was quantified with imageJ.

### Gene array

Cells were lysed with TRIzol and RNA isolated per manufacturer’s protocol. RNA was further purified by DNase and RNeasy Mini Kit (Qiagen). cDNA was generated using the High-Capacity cDNA Reverse Transcription Kit (Applied Biosystems). Mouse lipid regulated gene expression levels were determined using the Taqman Array Plates from Applied Biosystems per manufacturer’s protocol using the Applied Biosystems 7000 sequence detection system. The relative expression of genes were calculated against four housekeeping genes using the 2^−ΔΔCT^ method.

### Statistical analysis

The data are expressed as means ± SEM. Significant differences between two groups were determined by unpaired student’s t-test (2-tailed) and differences among multiple groups were determined by 1-way ANOVA with a Tukey post-hoc multiple comparison test. A *P* value of < 0.05 was accepted as statistically significant.

## Results

### CD36 promotes lung fibrosis in response to oxPL

**Fig. 1:**
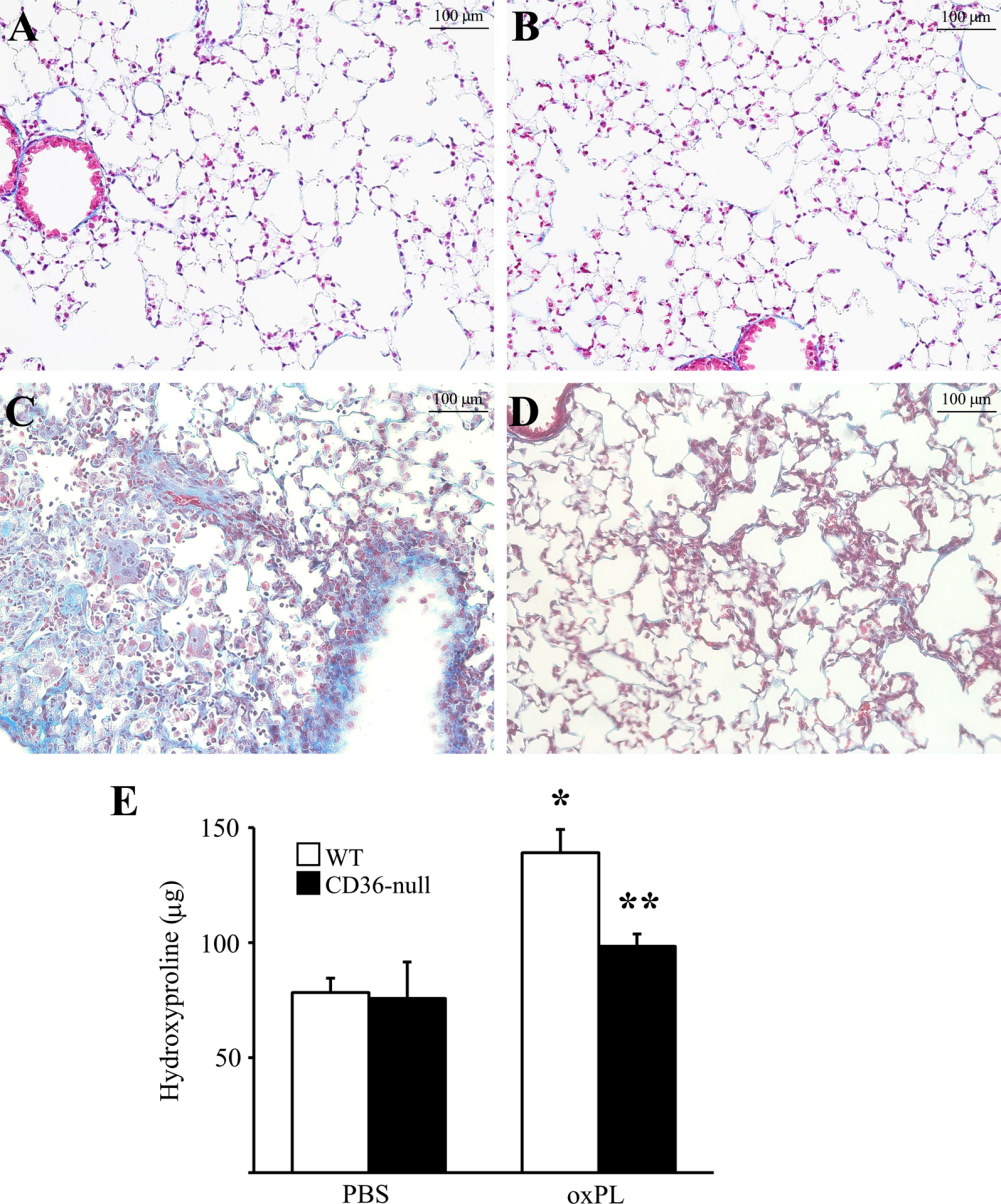
oxPL induces lung fibrosis in a CD36-dependent manner. Trichome stained lung sections (200x) from WT (**A**, **C**) or CD36-null (**B**, **D**) mice 14 days after intratracheal delivery of PBS (**A**, **B**) or oxPL (POVPC at 10 µg/gram) (**C**, **D**). **E** Hydroxyproline assay of lungs from WT and CD36-null mice 14 days after intratracheal instillation of oxPL or PBS control. n = 8–20/group. *p < 0.01 compared to WT mice treated with PBS. ** p < 0.05 compared to WT mice treated with oxPL

We have previously shown that delivery of apoptotic AEC2s into the uninjured lungs of mice induces fibrosis in a CD36-dependent manner [15]. Injured/apoptotic AEC2s can potentially cause fibrosis through several mechanisms including release of pro-fibrotic mediators, the formation of apoptotic bodies, and/or release of oxPL. Notably CD36 has been implicated in efferocytosis of apoptotic bodies [29] and the scavenging of oxPL [30]. In the current study, we sought to determine the extent to which the profibrotic activity of free, extracellular oxPL drives fibrosis in a CD36-dependent manner [30]. Although there are multiple forms of oxPL, based on a recent report [16], we chose to administer a single dose of purified POVPC (at 10 µg/gram body weight) into the lungs of healthy WT and CD36-null adult mice. Importantly, POVPC is an oxidized derivative of phosphatidylcholine, a major constituent of surfactant lipoprotein. Consistent with the prior study [16], we found that oxPL instillation induces robust fibrosis by day 14 in WT mice as evidenced by increased interstitial collagen deposition (assessed by histopathological observation of trichrome-stained lungs; Fig. 1A–D) and Additional file 1: Figure S1) and confirmed by an increase in total lung collagen content (assessed by hydroxyproline quantification; Fig. 1E). In contrast, CD36-null mice developed a significantly attenuated fibrotic response to oxPL exposure as evidenced by reduced collagen deposition and a decrease in total lung collagen content.

### CD36 does not alter common indicators of acute lung injury in response to oxPL

**Fig. 2:**
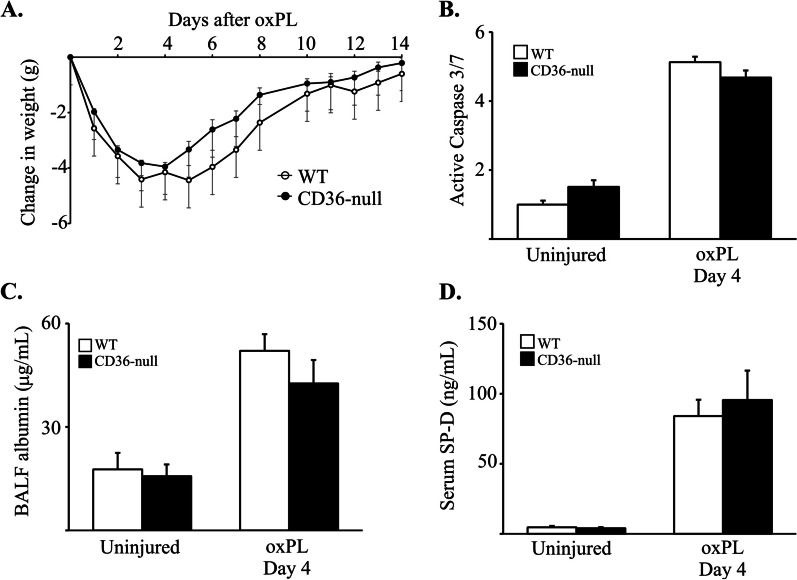
CD36 does not affect acute lung injury after oxPL. **A** WT and CD36-null mice were injured with oxPL (POVPC at 10 µg/gram) and change in weight determined over a 14 day period. N = 36. **B** Lung levels of active caspase 3/7 4 days after oxPL. N = 4–6 per group. **C** BAL fluid albumin 4 days after oxPL. N = 4–6 per group. **D** Serum levels of AEC2-marker surfactant protein D 4 days after oxPL. N = 5 per group

After determining that CD36 is required for the development of oxPL-induced fibrosis, we next assessed whether CD36 impacted the acute injury response to oxPL administration. We used complimentary endpoints to evaluate acute lung injury (relative to control mice exposed to PBS; Fig. 2). Measurements included weight loss, caspase 3/7 activation, alveolar protein leak, and serum surfactant protein-D (SP-D) levels following a single administration of oxPL (relative to control mice exposed to PBS; Fig. 2). We observed that WT mice exposed to oxPL lost weight (relative to PBS-exposed control mice) for 4 to 5 days following exposure before beginning to recover (Fig. 2A). Weight loss was associated with increases in whole lung caspase 3/7 activation (Fig. 2B), alveolar leak (Fig. 2C), serum SP-D levels (Fig. 2D) and BAL cell count (Additional file 1: Figure S2). In CD36-null mice, each marker of acute lung injury was comparable to the oxPL-exposed WT group. Lung sections from WT and CD36-null mice were also visualized for pro-surfactant protein C (SP-C) and TUNEL staining (Additional file 1: Figure S3). We found that POVPC induced TUNEL positive cells in both WT and CD36-null mice and a subset of the TUNEL-positive cells were also SP-C-positive. Thus, our in vivo findings indicate that the presence/absence of CD36 expression did not alter local or systemic indicators of acute lung injury in response to oxPL exposure.

### CD36 alters resident and recruited macrophage response to oxPL

**Fig. 3:**
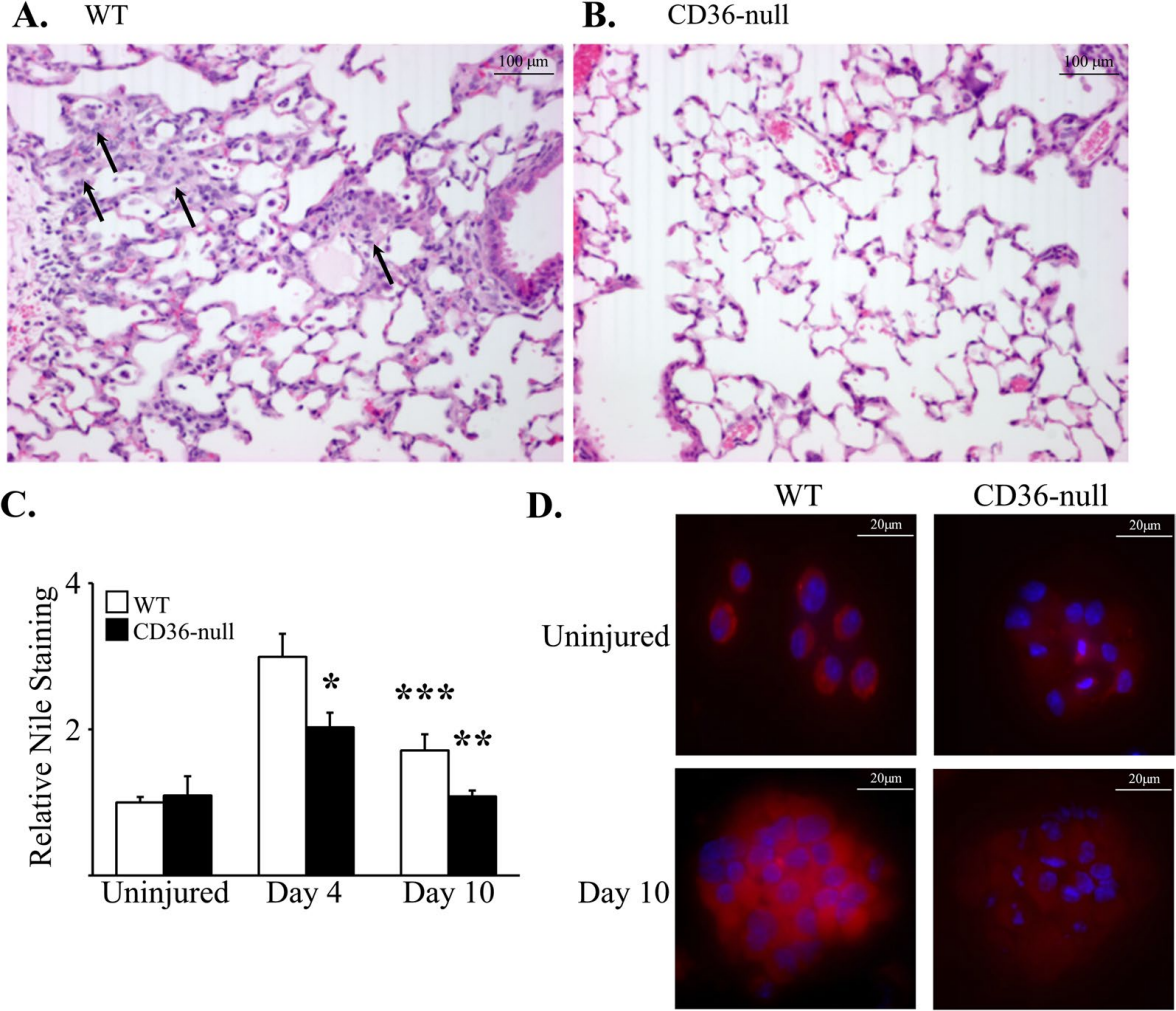
Lipid uptake by BAL cells and BAL TGFβ concentration are regulated by CD36. **A**, **B** Four days after intrapulmonary delivery of oxPL (POVPC at 10 µg/gram) lung sections were visualized by H&E staining (200x). WT mice (**A**) develop alveolar accumulation of large lipid laden foamy macrophages (arrows). CD36-null mice have less accumulation of foamy macrophages (**B**). **C** Lipid accumulation determined by nile red staining of BAL cells from WT and CD36-null mice at several timepoints after treatment with PBS or oxPL (POVPC 10 µg/gram). N = 8–12 per group. *p < 0.01 compared to WT mice 4 days after oxPL. **p < 0.01 compared to WT mice 10 days after oxPL. ***p < 0.05 compared to uninjured WT mice. **D** Representative images (400x) of nile red stained BAL cells from uninjured WT and CD36-null mice 10 days after oxPL injury

The absence of data implicating CD36 in the acute injury response to oxPL motivated studies to assess the impact of CD36 during later time points after oxPL-induced injury. We have previously shown that lung macrophage accumulation promotes fibrosis in response to targeted AEC2 injury [31–33], and others have shown that AEC2 injury is associated with oxPL release [16, 34]. Therefore, we explored the hypothesis that the profibrotic response to AEC2 injury might be modulated by CD36-dependent scavenging of oxPL by lung macrophages. This hypothesis is supported by several prior observations including: (1) lung macrophages express high levels of CD36 [6, 7] and (2) lipid laden, “foamy” macrophages consistently accumulate within fibrotic tissue [35]. To test this hypothesis, WT and CD36-null mice were administered intrapulmonary oxPL (versus PBS for control groups). We observed that instillation of oxPL induced an increased number of enlarged cells with macrophage morphology within the alveolar space in WT mice (Fig. 3A). These cells were less apparent in CD36-null mice (Fig. 3B). To follow-up on this observation, we isolated bronchoalveolar lavage (BAL) cells and measured their lipid content by nile red staining (Fig. 3C, D). We observed that the intrapulmonary instillation of oxPL resulted in an increase in nile red staining in WT BAL cells with a peak at day 4. Importantly, levels of nile red staining remained above baseline at day 10 in the BAL cells from WT mice (Fig. 3). In contrast, BAL cells isolated from oxPL-injured CD36-null mice exhibited significantly less nile red staining. Increased levels of lipid within BAL cells in WT mice 4 days after POVPC treatment was further confirmed by bodipy staining [36, 37] and flow cytometry analysis for bodipy also supported increased lipid accumulation within BAL cells from WT mice injured with POVPC when compared to the CD36-null group (Additional file 1: Figure S4). Finally, lung sections from WT and CD36-null mice 4 days after POVPC treatment (or uninjured) were analyzed by staining for bodipy and immunostaining for CD36 and CD45 (Additional file 1: Figure S5, S6). Lung sections from WT mice injured with POVPC demonstrated cells that stained positive for bodipy (compared to both CD36-null mice treated with POVPC and to uninjured mice). A subset of these bodipy positive cells were also positive for CD36 and CD45.

**Fig. 4:**
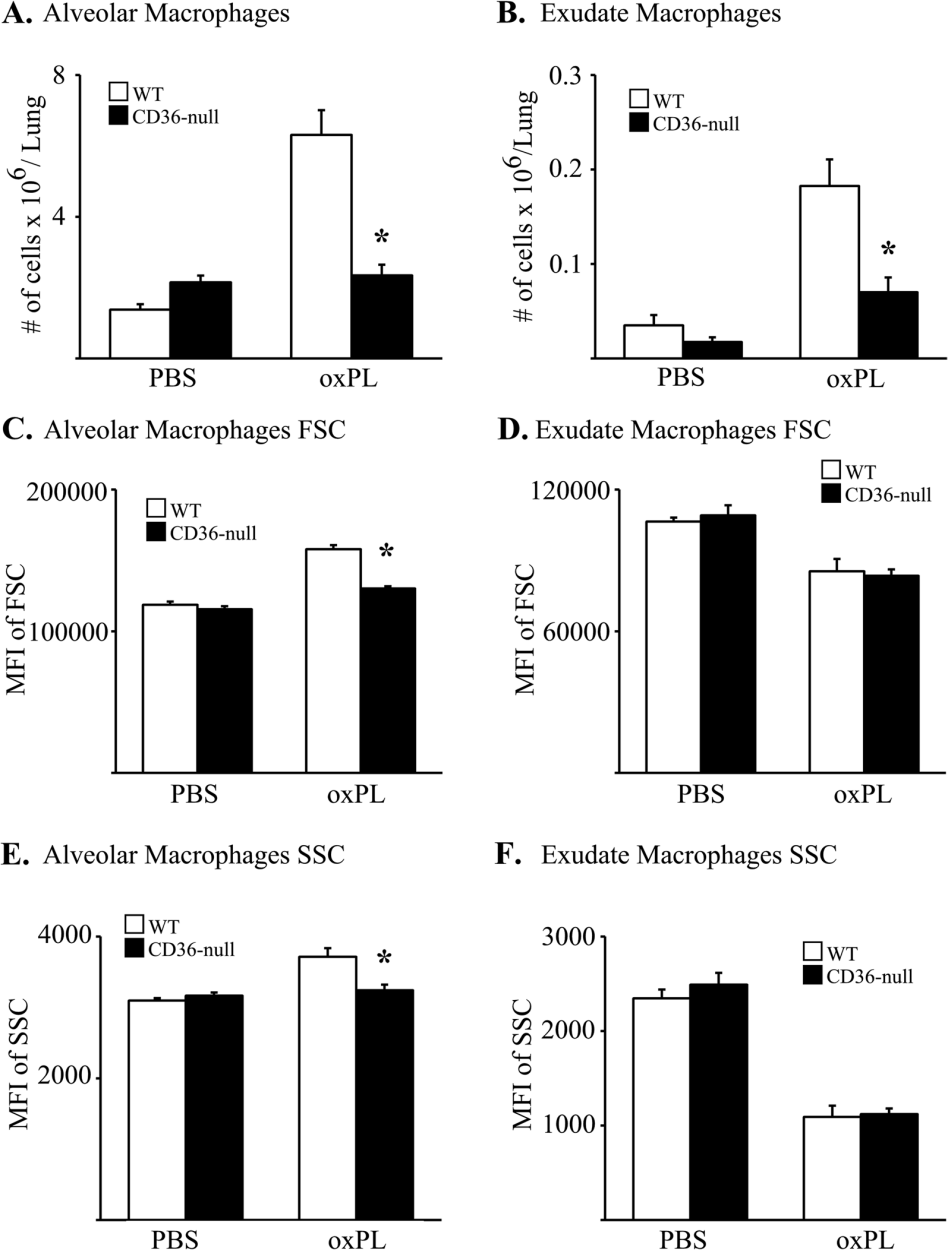
CD36 regulates accumulation macrophages after oxPL injury. CD36-null mice have less accumulation of alveolar macrophages (**A**) and exudate macrophages (**B**) 10 days after treatment with oxPL (POVPC 10 µg/gram). N = 4–12 per group. *p < 0.01 compared to WT mice treated with oxPL. **C** Relative size, assessed by mean fluorescent intensity of forward scatter (MFI of FSC), of alveolar macrophages. **D** Relative size of exudate macrophages. **E** Cell granularity, assessed by mean fluorescent intensity of side scatter (MFI of SSC), of alveolar macrophages. **F** Cell granularity of exudate macrophage. N = 4 per group. *p < 0.05 compared to WT mice treated with PBS. **p < 0.05 compared to WT mice treated with oxPL

We further characterized the macrophage response to oxPL instillation in WT and CD36-null mice using flow cytometric analysis and established gating schemes [27, 31, 32, 38] (Additional file 1: Figure S7). We specifically enumerated and phenotyped resident alveolar macrophages (AM) and recruited exudate macrophages (ExM) in the lungs of mice at day 10 post oxPL exposure (relative to control mice exposed to PBS; Fig. 4 and Additional file 1: Figure S8). Results demonstrate a robust increase in the number of AM (Fig. 4A) and ExM (Fig. 4B) in oxPL-exposed WT mice. In contrast, significantly fewer AM and ExM accumulated in CD36-null mice after oxPL instillation. The number of Ly-6C^high^ monocytes, precursors of ExM, was also reduced in the oxPL-injured CD36-null group (data not shown).

In light of the increased nile red staining in WT BAL cells on day 10 post-oxPL administration, we reasoned that the quantity of oxPL within the lung macrophage populations might be assessed by measures of forward scatter (an indicator of cell size) and side scatter (an indicator of cell granularity) using flow cytometric analysis, although this method best characterizes immune cells in the blood. With this approach, we identified a significant increase in forward scatter (Fig. 4C) and side scatter (Fig. 4E) in the AM population isolated from the lungs of WT mice at day 10 post-oxPL which was not observed in the AM population from the comparably treated CD36-null mice. Interestingly, forward scatter (Fig. 4D) and side scatter (Fig. 4F) was not increase in the ExM of the oxPL-exposed WT mice relative to either the PBS-exposed WT mice or the oxPL-exposed CD36 null mice. This finding supports the possibility that the non-resident ExM were recruited into the lung after the administered oxPL had been largely taken up by the resident AM.

**Fig. 5:**
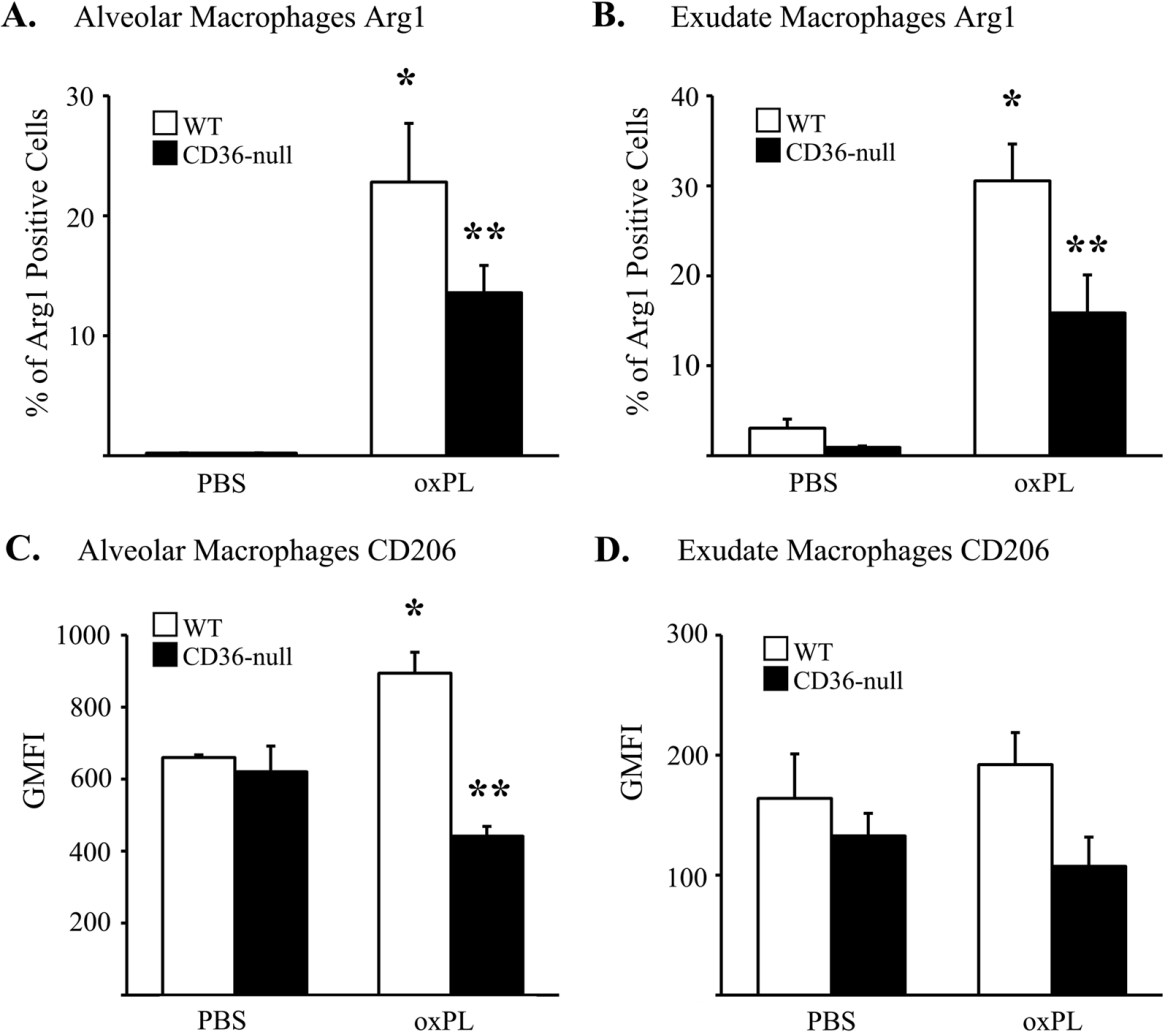
CD36 regulates macrophage expression of pro-fibrotic markers after oxPL injury. Expression of arginase 1 (Arg1) and CD206 on alveolar and exudate macrophages isolated from WT and CD36-null mice 10 days after oxPL (POVPC at 10 µg/gram) determined by flow cytometry. **A** Percentage of alveolar macrophages expressing Arg1. **B** Percentage of exudate macrophages expressing Arg1. **C** Alveolar macrophage expression of CD206 expressed as geometric mean fluorescent intensity (GMFI). **D** Exudate macrophage expression of CD206. N = 4 per group. *p < 0.05 compared to WT mice treated with PBS. **p < 0.05 compared to WT mice treated with oxPL

We previously showed that AM and ExM accumulating in response to selective AEC2 injury express a phenotype that can be considered “alternatively-activated” and/or “profibrotic” [31, 32]. To assess whether this phenotype might be modulated by interactions between CD36 and oxPL, we used flow cytometric analysis to assess expression of CD206 and arginase (markers of alternative activation) on AM and ExM on day 10 post-injury in WT and CD36-null mice (relative to control mice; Fig. 5 and Additional file 1: Figure S9). Results in the WT group demonstrated that oxPL exposure was associated with an increase in CD206 (Fig. 5C) and arginase (Fig. 5A) expression in AMs, and a significant increase in arginase (Fig. 5B) and a trend toward an increase CD206 (Fig. 5D) in ExMs (relative to WT mice exposed to PBS). In contrast, there was less expression of both markers in CD36-null AMs (Fig. 5A, B). Arginase expression was also reduced in ExMs isolated from the lungs of CD36-null mice exposed to oxPL (relative to WT mice, Fig. 5C), and we observed an insignificant decrease in the expression of CD206 in CD36-null ExMs compared to WT ExMs (Fig. 5D, p = 0.18). Amongst two other macrophage activation markers, expression of CD80 was also reduced in the AM and ExM of CD36-null mice whereas iNOS expression was low (< 1%) in macrophages from both strains of mice and did not vary with oxPL exposure (data not shown).

### CD36 promotes TGFβ production by macrophages in response to oxPL

**Fig. 6:**
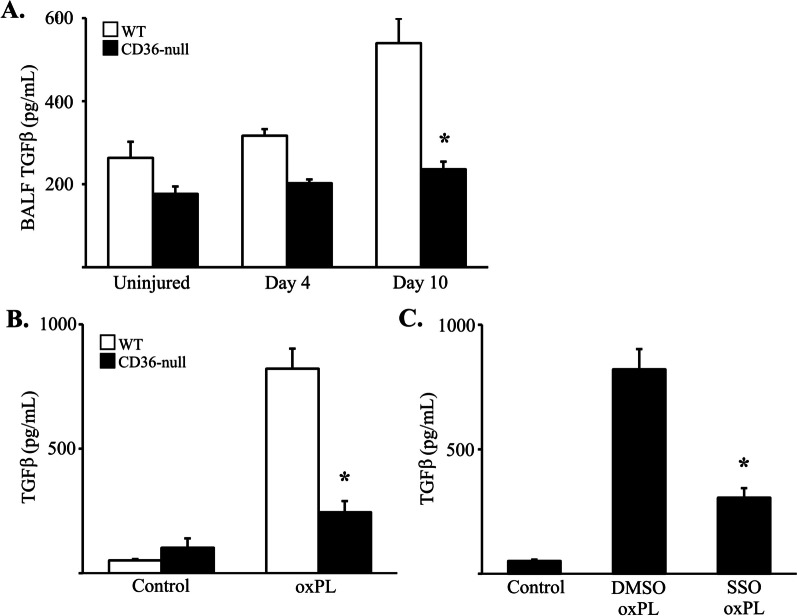
CD36 regulates TGFβ production by macrophages. **A** TGFβ ELISA of BAL fluid from WT and CD36-null mice at several timepoints after treatment with PBS or oxPL (POVPC at 10 µg/gram). N = 4–6 per group.,*p < 0.01 compared to WT mice treated with PBS. **B** TGFβ ELISA of conditioned media from WT and CD36-null bone marrow derived macrophages (BMM) treated with or without oxPL (POVPC at 10 µg/ml). N = 4–6 per group.,*p < 0.01 compared to WT mice treated with PBS. **C** TGFβ ELISA of conditioned media from WT BMMs treated with 100 µM SSO (vs. DMSO vehicle control) and oxPL. N = 4–6 per group.,*p < 0.01 compared to BMMS treated with DMSO and oxPL

Macrophage efferocytosis of apoptotic AEC2 and uptake of oxidized lipoproteins has been shown to promote expression of the profibrotic growth factor, TGFβ [39, 40]. Having shown that CD36 and oxPL exposure alters AM and ExM phenotype in this model, we next sought to determine whether CD36/oxPL interactions promote increased production of TGFβ. We first collected BAL fluid at day 4 and 10 from the lungs of WT and CD36-null mice exposed to oxPL (or PBS) and measured TGFβ levels (by ELISA; Fig. 6A). We observed that oxPL administration caused an increase in TGFβ in WT mice that was modest at day 4 but more robust by day 10. In contrast, CD36-null mice had minimal increase in TGFβ protein expression.

To further interrogate this pathway, we performed in vitro experiments to assess TGFβ production in macrophages following exposure to oxPL. Bone marrow derived macrophages from WT and CD36-null mice were isolated and cultured. Macrophages were then exposed to oxPL (POVPC at 10 µg/ml) versus vehicle, and TGFβ was quantified (by ELISA) in the conditioned media after 24 h (Fig. 6B). Results demonstrate a marked increase in TGFβ production by macrophages from WT mice in response to oxPL. In contrast, TGFβ production was attenuated in macrophages obtained from CD36-null mice. To further confirm that CD36 mediates TGFβ production in response to oxPL, we pretreated WT macrophages for 1 h with SSO, a CD36 inhibitor, and assessed TGFβ protein levels by ELISA in conditioned media at 24-h post oxPL exposure (Fig. 6C). We found that TGFβ production was significantly inhibited in oxPL-exposed macrophages by SSO treatment compared to vehicle. We next interrogated whether the CD36-mediated induction of TGFβ production was specific to oxPL or whether another pro-fibrotic stimulus would also induce TGFβ in a CD36-dependent manner. Prior reports suggested that macrophages and other cells are activated in response to in vitro bleomycin exposure at a dose range of 5 to 100 mU/ml [41, 42]. Consistent with these prior reports, we found that macrophages treated bleomycin (5 mU/ml) had a modest upregulation of TGFβ expression but as opposed to response of cells treated with POVPC, the upregulation in TGFβ was similar between WT and CD36-null cells (Additional file 1: Figure S10). Lower doses of bleomycin failed to induce a TGFβ response while higher doses were toxic and killed both WT and CD36-null cells. Collectively, these data support our hypothesis that CD36-mediated uptake and accumulation of oxPL in lung macrophages stimulates their production of TGFβ in the alveolar space and thereby potentiates the development of pulmonary fibrosis.

### CD36 mediated TGFβ production by macrophages in response to oxPL is dependent on Lyn kinase activation

**Fig. 7:**
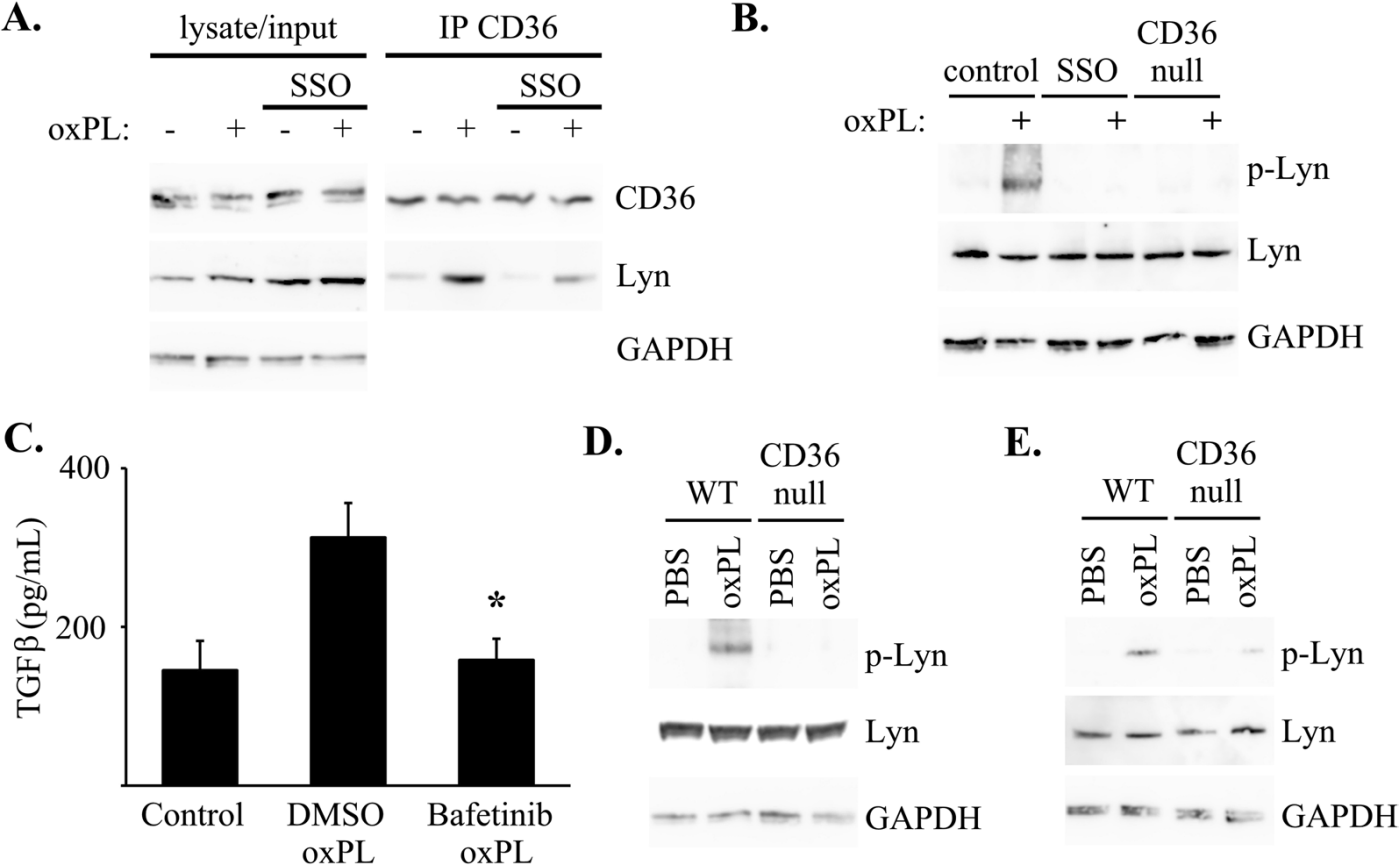
CD36-dependent TGFβ production in response to oxPL is mediated by Lyn kinase. **A** Lysate from bone marrow derived macrophages (BMM) treated with 100 µM SSO (vs. DMSO vehicle control) and oxPL (POVPC at 10 µg/ml) were immunoprecipitated for CD36 and analyzed by immunoblot for CD36 and Lyn kinase. **B** WT BMMs treated with 100 µM SSO (vs. DMSO vehicle control) and CD36-null BMM were treated with oxPL (POVPC at 10 µg/ml) were analyzed for levels of Lyn and phosphorylated Lyn (p-Lyn). **C** TGFβ ELISA of conditioned media from WT BMMs treated with 10 µM Bafetinib (vs. DMSO vehicle control) and oxPL. N = 4–6 per group,*p < 0.01 compared to BMMS treated with DMSO and oxPL. **D**, **E** Four days after WT and CD36-null mice were injured with POVPC (or PBS control) lysate from lungs (**D**) or BAL cells (**E**) were analyzed for Lyn and p-Lyn by immunoblot. Images shown are cropped from the the original image files

Prior studies implicate Lyn kinase, a member of the src family of tyrosine kinases, in macrophage acquisition of the foam cell phenotype in the context of atherosclerosis [13, 43]. To assess whether TGFβ production by lung macrophages in response to oxPL involves Lyn kinase signaling, we assessed for the formation of CD36/Lyn complexes in WT bone marrow derived macrophages following exposure to oxPL (relative to control mice; Fig. 7A and Additional file 1: Figure S11). A subset of WT bone marrow derived macrophages were pretreated with SSO (or vehicle control) 1 h prior to oxPL exposure (as described above). Two hours later, macrophage lysates were immunoprecipitated for CD36 and assessed for CD36/Lyn complexes (by immunoblot). CD36/Lyn complexes were identified in the oxPL-exposed WT macrophages whereas pretreatment of with SSO (CD36 inhibitor) diminished CD36/Lyn complex formation.

We next determined whether CD36/Lyn complexes induced by oxPL exposure resulted in Lyn activation as determined by phosphorylation at Tyr396 (Fig. 7B and Additional file 1: Figure S11). WT and CD36-null bone marrow-derived macrophages were isolated, pretreated with or without SSO, and exposed to oxPL. After 2 h, macrophage lysates were analyzed by immunoblot for phospho-Tyr396-Lyn and total Lyn. Lysates from oxPL-exposed WT macrophages demonstrated a marked increase in Lyn phosphorylation whereas Lyn phosphorylation was comparatively attenuated in lysates obtained from CD36-null macrophages and from WT macrophages pre-treated with SSO.

To further establish the link between CD36-mediated activation of the Lyn kinase signaling pathway and TGF-β production in response to oxPL, bone-marrow derived macrophages obtained from WT mice were pretreated for 1 h with a Lyn inhibitor, Bafetinib (10 µM), or vehicle control prior to exposure to oxPL. After 24 h, the levels of TGFβ in conditioned media was analyzed (by ELISA; Fig. 7C). TGF-β production was markedly increased following oxPL exposure in control macrophages whereas pretreatment with Bafetinib markedly diminished TGFβ production.

To determine whether the CD36-dependent Lyn activation observed in our in vitro oxPL exposure experiments was recapitulated in vivo, we harvested lungs and BAL cells from WT and CD36-null mice 4 days after oxPL administration. Activation of Lyn was assessed by immunoblot for phospho-Lyn and we found in both whole lung lysates (Fig. 7D and Additional file 1: Figure S11) and BAL cell lysates (Fig. 7E and Additional file 1: Figure S11) that Lyn activation was markedly increased in oxPL-exposed WT mice whereas Lyn phosphorylation was nearly absent in CD36-null mice. Together, our in vitro and in vivo results establish that Lyn is a critical mediator of CD36-dependent TGFβ production by macrophages in response to oxPL.

Finally, we wanted to expand, beyond TGFβ, the macrophage response to oxPL. We treated bone marrow derived macrophages from WT and CD36-null mice with or without oxPL as before. After 24 h RNA was isolated and analyzed by a the Mouse Lipid Regulated Genes targeted array (Additional file 1: Figure S12 and Additional file 2: Table S1).

## Discussion

CD36 is a multifunctional receptor that has been implicated as a key mediator of tissue fibrosis in multiple organs. In this regard, we previously demonstrated that CD36 is required for pulmonary fibrosis following the instillation of apoptotic AEC2 cells into the lungs of healthy mice [15]. In the present study, considering the role of CD36 as a known scavenger receptor for oxPL and that apoptotic AEC2 cells are a rich source for oxPL, we sought to determine whether CD36 promotes the development of lung fibrosis in a novel murine model of intrapulmonary oxPL administration. For these studies we wanted to begin with a specific injury with POVPC, however in future studies it will be interesting to examine the diversity of oxidized lipids produced following injury/apoptosis of AEC2. Our data, collected at several time points, suggest that CD36-mediated accumulation of lipid within macrophages is a critical mediator of fibrosis. WT and CD36-null mice exhibited similar levels of acute injury 4 days after oxPL. However, WT BAL cells had greater levels of lipid accumulation (compared to CD36-null BAL cells) at this time point which we hypothesize drives subsequent changes in macrophage accumulation and expression of pro-fibrotic TGFβ on day 10 after oxPL injury. These changes then result in differences in fibrosis measured 14 days after oxPL. We found that mice expressing CD36 (when compared to CD36-null mice) had significantly worse fibrosis in response to oxPL and that the scarring was associated with the accumulation of an increased number of lung macrophages. CD36 also accentuated the accrual of oxPL within lung macrophages and promoted the acquisition of a pro-fibrotic phenotypic alteration marked by TGFβ expression. Finally, we identified a src family kinase signaling pathway that linked macrophage oxPL uptake with profibrotic macrophage gene expression. Together, these observations elucidate a novel pathogenic mechanism of profibrotic macrophage activation via CD36 with several potential therapeutic targets.

### CD36 promotes tissue fibrosis

Our central finding in the present study is the requirement of CD36 expression for the development of lung fibrosis following oxPL administration. We demonstrate using lung hydroxyproline and histology that CD36 null mice develop significantly less fibrosis than WT control animals on day 14 post-oxPL instillation. These results add to a growing literature that implicates CD36 in tissue fibrosis in multiple organs including the liver, kidney and lung. For example, the downregulation of CD36 expression in the liver by MicroRNA-29 led to an attenuation of high fat diet induced steatosis along with a reduction in the expression of fibrotic markers including Col1a1 [44]. Furthermore, in the unilateral ureteral obstruction model of kidney injury, CD36 expression was associated with increased levels of lipid peroxide accumulation within macrophages, and this cellular alteration resulted in increased tubulointerstitial fibrosis and worse kidney function [45]. With respect to the lung, our current results compliment the findings of two other reports that implicate CD36 in pulmonary fibrosis. Specifically, CD36 deficient mice were found to be resistant to bleomycin-induced fibrosis and our prior study demonstrated that CD36 deficiency attenuated the fibrotic response induced by the intrapulmonary instillation of apoptotic AEC2 cells [6, 15]. CD36 has also been implicated in lung fibrosis induced by silica [7]. The observation that CD36 plays a profibrotic role in multiple different organs following disparate insults implicates this molecule as an attractive therapeutic target for a variety of scarring disorders.

### CD36-mediated macrophage accumulation

In the present study, we found that oxPL-induced injury results in a CD36-dependent increase in the number of macrophages in the lungs (Fig. 4). Specifically, on day 10 post-oxPL, the number of resident alveolar and recruited exudate macrophages was over twofold greater in mice expressing CD36 compared to the CD36-null group. This observation suggests a possible mechanism by which oxPL, via CD36, induces scarring, as recent evidence from our group and others implicates a causal role for the recruitment of bone marrow derived Ly-6C-high monocytes and exudate macrophages in the promotion of scarring following a variety of lung injuries [32, 46]. Particularly compelling are several studies that used different approaches to prevent the recruitment of Ly-6C-high monocytes and exudate macrophages into the lung following a fibrotic insult and found that this intervention attenuated scarring [46–48]. The contribution of resident alveolar macrophages to the pathogenesis of lung fibrosis is less clear, but as mentioned, this population was markedly expanded in CD36-expressing WT mice on day 10 following oxPL instillation. The mechanism by which CD36 promotes the accumulation of these macrophage populations following oxPL administration has yet to be fully elucidated. However, we hypothesize that, through its role as a scavenger receptor for oxPL, that CD36-mediated binding/uptake of oxidized phosphocholine by the different macrophage populations induces their expression of mitotic growth factors, chemotactic cytokines and/or affords protection against apoptotic stimuli.

### CD36-mediated foam cell formation

Using several different techniques, we found that CD36 expression mediated the formation of large, lipid-laden macrophages, i.e. “foam cells”, following oxPL instillation. Specifically, we found histopathologic evidence of an increased number of enlarged intraalveolar cells with macrophage morphology in WT mice on day 4 and day 14 post oxPL exposure (Figs. 1, 3). We also observed that lung macrophages isolated from the lavage fluid of CD36-expressing WT mice on days 4 and 10 post-oxPL exhibited increased nile red staining. In contrast, few intraalveolar enlarged cells were identified in lung sections obtained from oxPL-exposed CD36-null mice and lung macrophages from the CD36-null animals had attenuated nile red staining. Our flow cytometry data also suggest that the alveolar macrophage population in the WT group had increased lipid accumulation in that they were larger (increased forward-scatter) and more granular (increased side scatter) than the CD36-null alveolar macrophages.

The lipid-laden macrophage phenotype observed in our analyses is akin to the foam cell phenotype described in atherosclerotic lesions. The function of foam cells in the context of cardiovascular disease has been studied extensively, but their contribution to disease pathogenesis remains controversial and incompletely defined. Some reports suggest that foam cells exhibit a M1/pro-inflammatory phenotype while others suggest that foam cells exhibit an M2/anti-inflammatory phenotype [49, 50]. Recently, several studies have reported that the foam cell phenotype is associated with the upregulation of pro-fibrotic cytokines which may be important in the formation and stability of the atherosclerotic plaque [35]. The role of lipid-laden macrophages/foam cells in lung fibrosis is less well studied as compared to atherosclerosis. However, several observations implicate this macrophage phenotype in lung fibrogenesis in both mouse models and in patients. For example, Romero and colleagues identified lipid-laden macrophages as late as day 14 in the alveolar compartment of mice following single dose bleomycin [16]. Similarly, lipid-laden macrophages were observed in the lungs of rats on day 28 following injury with nitrogen mustard [51] and in the airways of mice with sustained club cell injury [38]. Notably, both bleomycin and nitrogen mustard injuries ultimately culminate in alveolar fibrosis whereas club cell injury resulted in small airway fibrosis. Furthermore, in silica-exposed patients, foam cell accumulation correlated with the severity of lung disease [34] and foam cells are identified in military personnel with deployment related respiratory disease [38]. Finally, the amount of apoA-I, a protein involved in cellular cholesterol, was found to be decreased in the lavage fluid of patients with IPF (compared to controls), and the levels were inversely correlated with the proportion of foamy AM isolated from IPF subjects [52].

Interestingly, although our nile red staining and histology cannot distinguish alveolar and exudate macrophages, our flow cytometry data indicate that the exudate macrophage population does not have the same degree of oxPL accumulation (i.e., these cells are smaller and less granular than the alveolar macrophages from WT mice). The significance of this disparity is unclear, but the difference may be the byproduct of the exudate macrophages entering the alveolar compartment at a time point that is more remote from the initial injury. It is also possible that this macrophage population expresses less CD36.

### CD36-mediated macrophage phenotypic alteration

In conjunction with the CD36-mediated increase in lung macrophage accumulation and lipid uptake, our data demonstrate that CD36 expression contributes to an alteration in cell phenotype following oxPL injury that includes an increased expression of CD206 and Arg1, markers of a profibrotic phenotype [53]. WT mice also exhibit increased TGF-β in their lavage fluid following oxPL installation, and our in vitro experiments confirm a direct link between oxPL exposure, macrophage CD36 expression/activity, and TGF-β expression. We expanded our analysis of genes regulated by oxPL and CD36 with an array focused on lipid regulated genes (Additional file 1: Figure S12, Additional file 2: Table S1). Several of these genes have also been implicated in fibrosis. Interestingly, CD36-null mice are protected from fibrosis despite reduced expression of Abca1 and Abcg1 by bone marrow derived macrophages. These lipid transporters are involved in efflux of lipid out of the cell and may be involved in limiting foam cell formation and fibrosis after injury [16, 54–56]. CD36-null cells have increased expression of LPL, IL-6 and IL1-β which have been implicated in inflammasome assembly during bleomycin induced pulmonary fibrosis [57, 58]. Although, the mechanism by which CD36 regulates expression of these genes remains poorly defined, we show that oxPL exposure induces an association of CD36 with the src kinase, Lyn, and that this association mediates Lyn phosphorylation. Importantly, inhibition of Lyn kinase with Bafetinib, a selective small molecule antagonist, downregulates the macrophage expression of TGFβ following oxPL exposure. In the context of lung fibrosis, although the recruitment of Ly-6c high monocytes and exudate macrophages is critical for lung fibrogenesis, not much is known about how these cells are activated nor the signaling pathway that links oxPL uptake with changes in cell phenotype. In cardiovascular disease, reports demonstrated an important role for CD36 signaling through Lyn kinase in establishing a foam cell phenotype in the context of atherosclerosis [13, 14]. Lyn kinase has also been shown to regulate other cellular phenotypes including proliferation and migration and has been implicated in hematologic malignances [59]. Although further studies are required to assess the role of Lyn kinase in mediating other macrophage phenotypic alterations such as Arg1 and CD206 expression, its regulation of TGFβ highlights its potential as a therapeutic target in lung fibrosis, and a number of small molecule inhibitors of Lyn kinase including Bafetinib, are in development.

### CD36-mediated efferocytosis versus oxPL uptake in lung fibrosis

The absence of CD36 expression following bleomycin injury led to the delayed clearance of apoptotic alveolar cells, and this observation led our laboratory to hypothesize that CD36-mediated efferocytosis of apoptotic cells by lung macrophages is a key step in fibrogenesis following lung injury. This hypothesis was supported by our data showing that CD36 was required for the development of lung fibrosis following apoptotic AEC2 administration [15]. Efferocytosis is a complex, coordinated process involving interaction between multiple molecules on the surface of the apoptotic body and the efferocytic cell [29, 60], and the specific mechanism by which CD36 translates apoptotic body binding and uptake into fibrosis is unclear. However, because apoptotic AEC2s are uniquely rich in oxPL as a byproduct of their critical function in surfactant lipoprotein production and CD36 is a scavenger receptor for long chain fatty acids including oxPL, it is plausible that oxPL is the key constituent of apoptotic cells that drives fibrosis. Our data that oxPL, in the form of oxidized phosphocholine, causes CD36-dependent fibrosis supports this possibility. Further studies are required to determine whether there are additional unique interactions between CD36 and apoptotic cells, independent of oxPL, that can also contribute to profibrotic phenotypic alterations. Further studies are also necessary to determine whether additional oxPL species other than oxidized phosphocholine can instigate scarring via CD36.

In summary, our findings in the present study elucidate a novel pathway by which CD36 promotes oxPL uptake by lung macrophages and thereby directly stimulates lung scarring through the accrual and pro-fibrotic activation of these cells that includes the expression of TGFβ. The translation of oxPL uptake by CD36 into TGFβ protein expression involves the activation of a src kinase family member, Lyn kinase. These observations identify several therapeutic targets in the context of lung fibrosis including oxPL, CD36 and an intracellular tyrosine kinase signaling cascade and motivate future studies to determine whether interrupting different steps of the oxPL-CD36 pathway holds therapeutic promise.

### Supplementary Information

**Additional file 1: Supplemental Figures 1-12**.

**Additional file 2: Supplemental Table 1.** Relative gene expression of lipid regulated genes by bone marrow derived macrophages from WT and CD36-null mice treated with or without POVPC (10 μg/mL).

## Data Availability

Not applicable.
