# Supplementary material for: CD36/Lyn kinase interactions within macrophages promotes pulmonary fibrosis in response to oxidized phospholipid
Source: Respir Res. 2023 Dec 14;24:314. doi: 10.1186/s12931-023-02629-6 (PMC10722854; doi:10.1186/s12931-023-02629-6)
Supplement: Supplementary file 1 — Additional file 1: Supplemental Figures 1-12. [file 12931_2023_2629_MOESM1_ESM.docx]

**
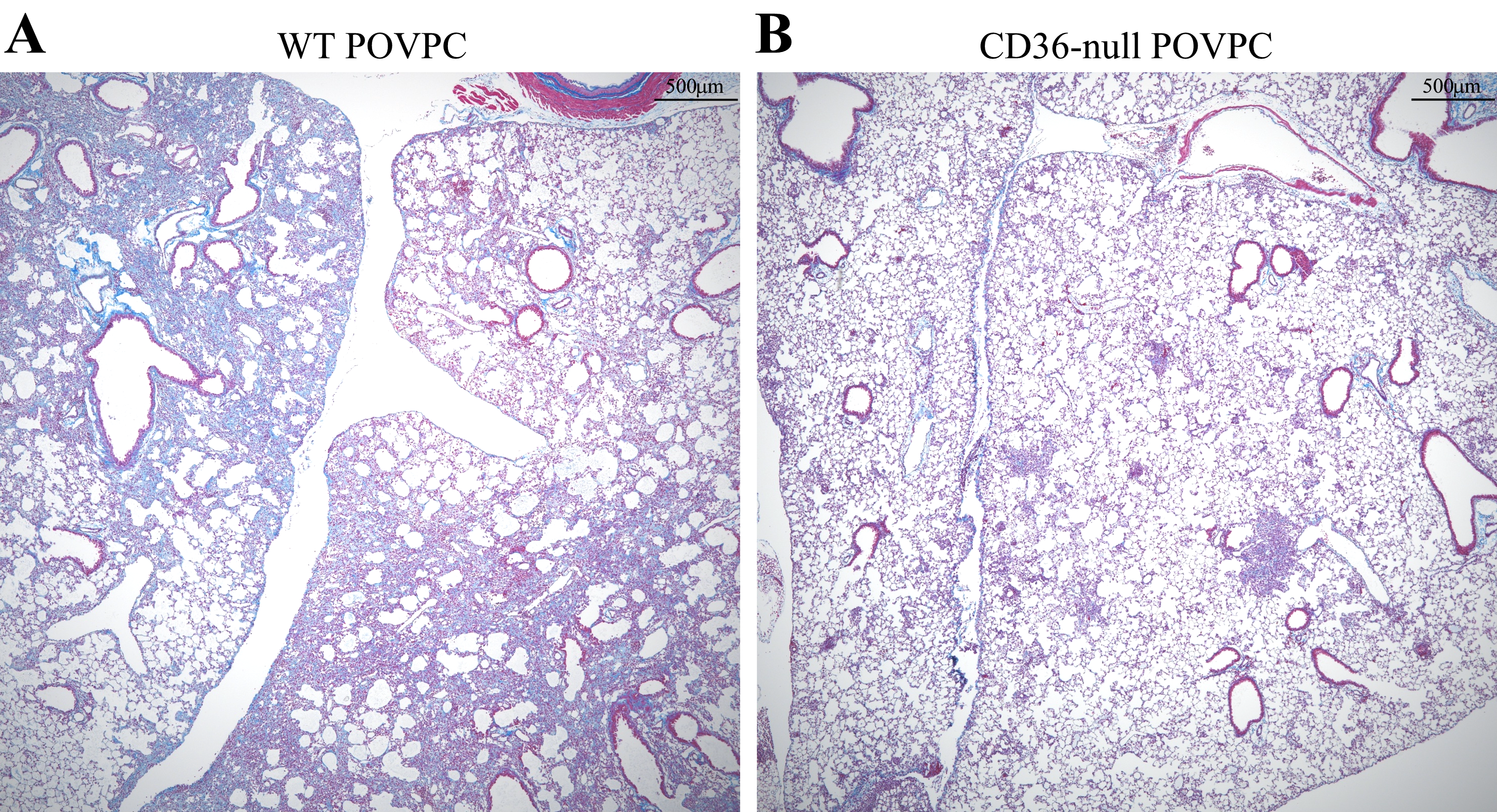
**

**Figure S1.** Trichrome stained lung sections (40x) from WT (**A**) or CD36-null (**B**) mice fourteen days after intratracheal delivery of oxPL (POVPC at 10 µg/gram).

**
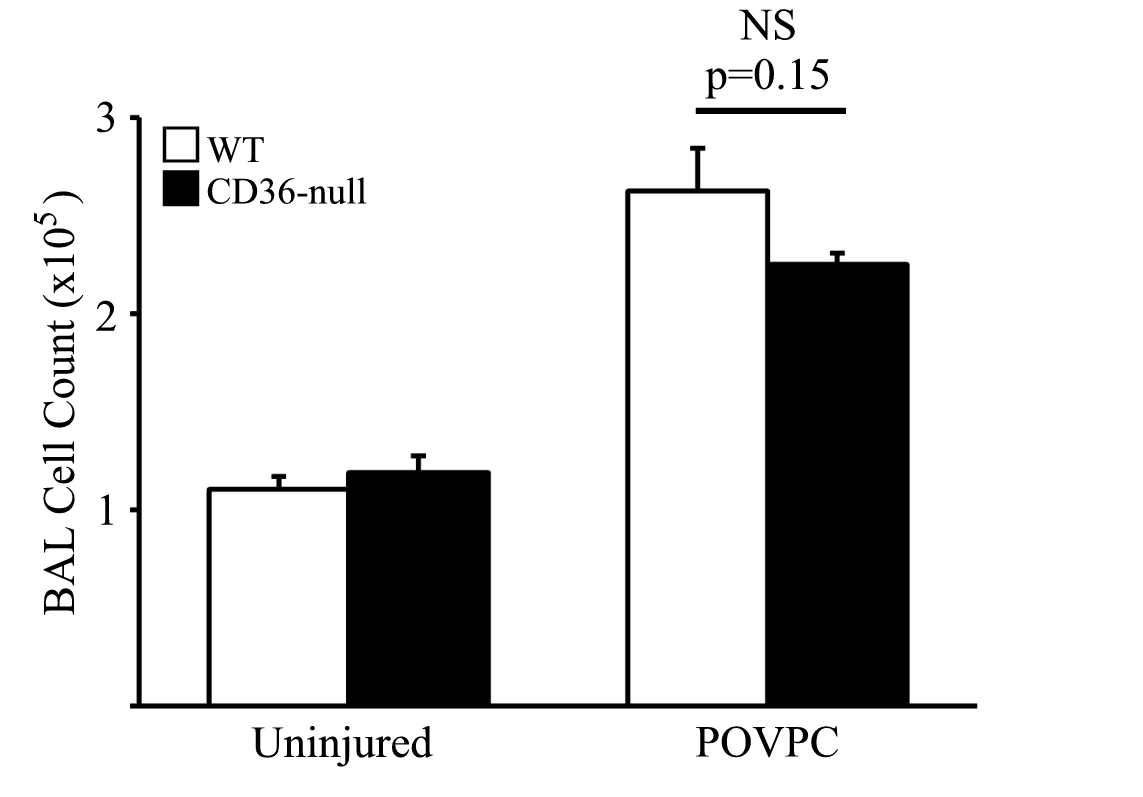
**

**Figure S2.** Four days after WT and CD36-null mice were injured with oxPL (POVPC at 10 µg/gram) total number of bronchoalveolar lavage cells was determined by counting on a hemocytometer. N=6 per group.


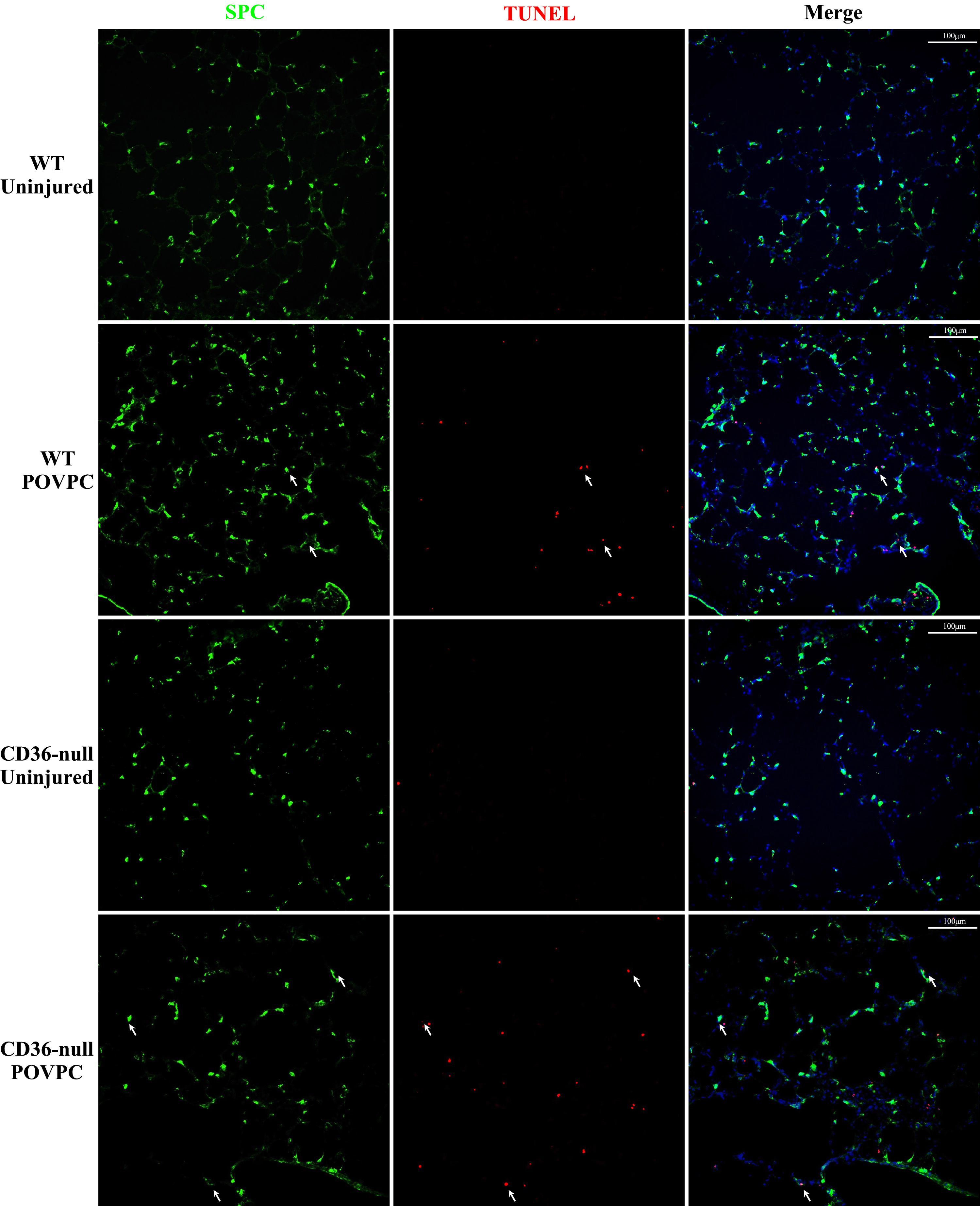


**Figure S3.** Four days after WT and CD36-null mice were injured with oxPL (POVPC at 10 µg/gram) lung sections were immunostained for prosurfactant protein-C (green) followed by TUNEL staining (red) and mounted in Dapi (blue) containing mounting media. Images are 200x.

**
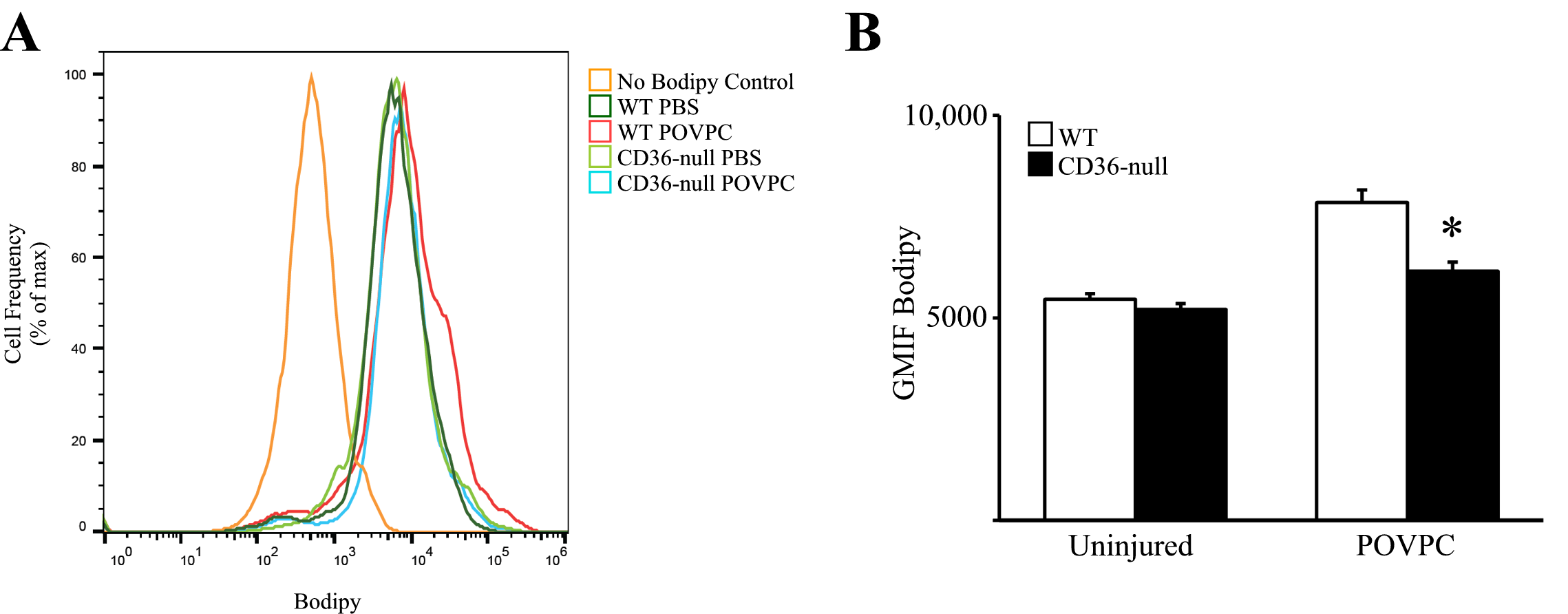
**

**Figure S4.** Four days after WT and CD36-null mice were injured with oxPL (POVPC at 10 µg/gram) BAL cells were stained with bodipy and analyzed by flow cytometry (A) and geometric mean fluorescent intensity (GMFI) determined. N=6, *p<0.05 compared to BAL cells from WT mice treated with POVPC

**
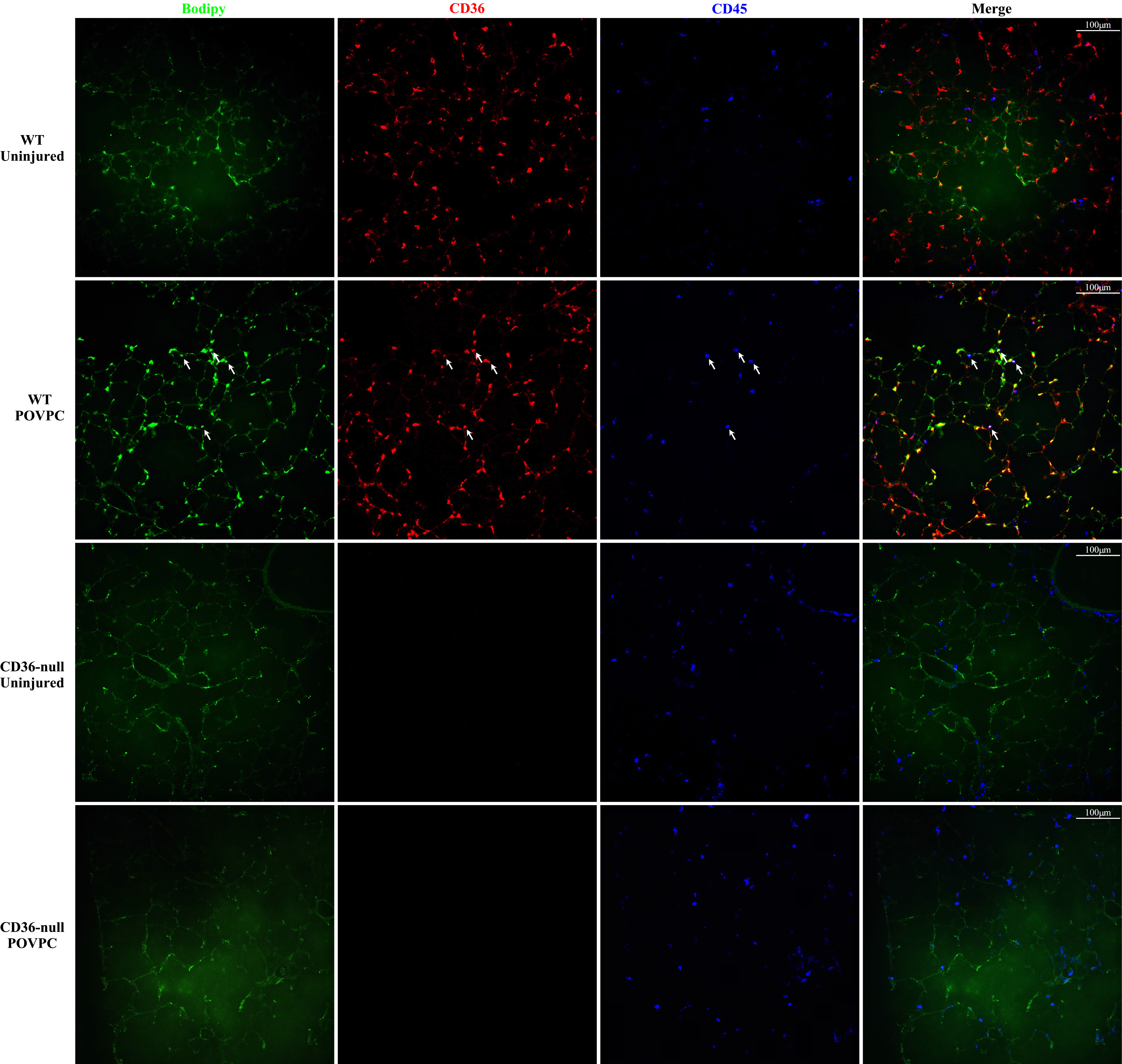
**

**Figure S5.** Four days after WT and CD36-null mice were injured with oxPL (POVPC at 10 µg/gram) and lung sections were stained with bodipy (green) and immunostained for CD36 (red) and CD45 (blue) and mounted in mounting media without Dapi. Several cells that are positive for bodipy, CD36 and CD45 are shown (arrows). Images are 200x.


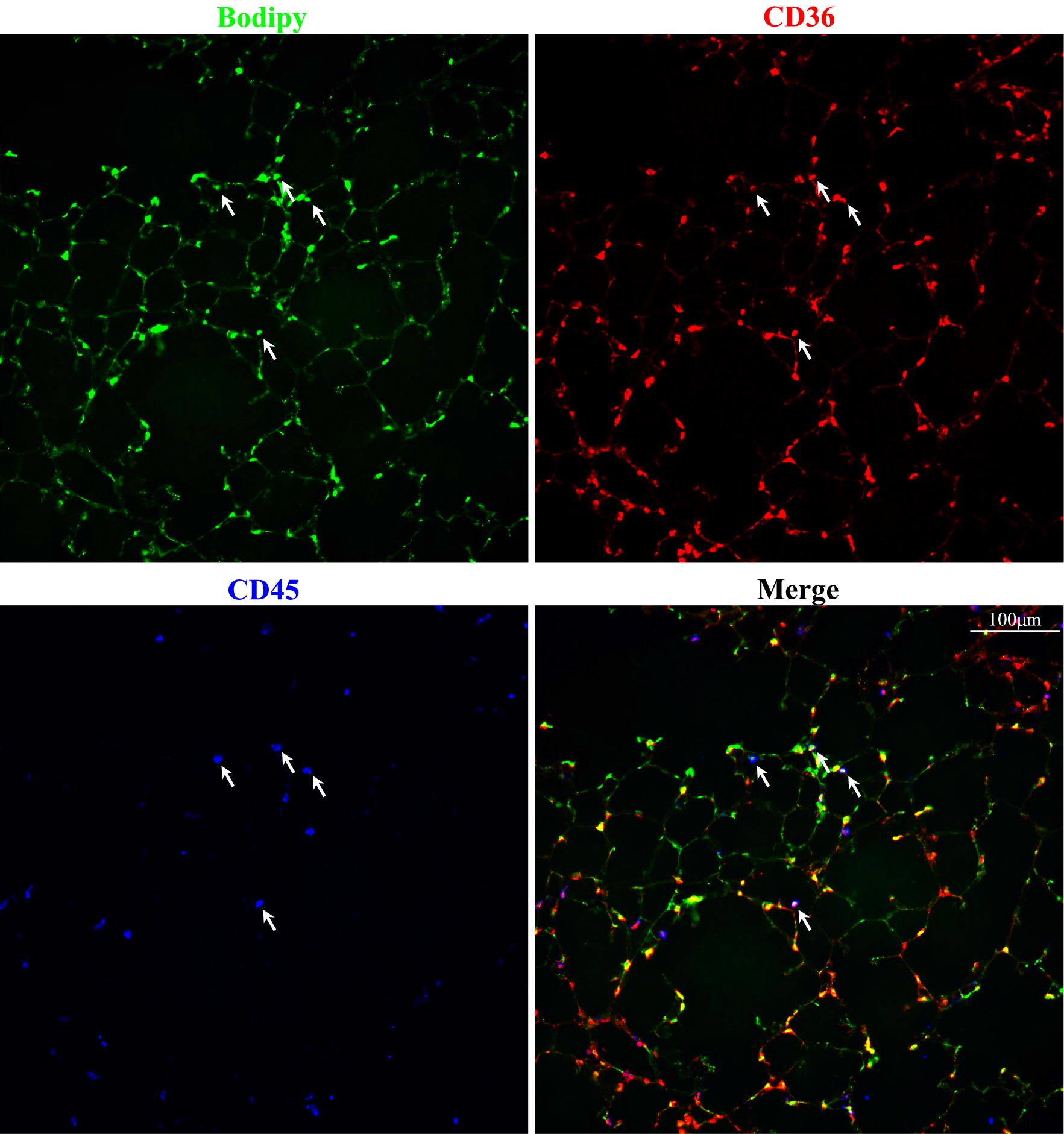


**Figure S6.** Larger images of lung sections from WT mice injured with oxPL shown in Supplement Figure 5. Lungs are stained with bodipy (green) and immunostained for CD36 (red) and CD45 (blue) and mounted in mounting media without Dapi. Several cells that are positive for bodipy, CD36 and CD45 are shown (arrows). Images are 200x.

**Figure S7.** Lung myeloid cell identification. Lung-derived single cell suspensions, obtained from C57BL/6 mice 10 days following oxPL exposure. Cells were stained using a viability dye and fluorochrome-conjugated antibodies targeting: CD45, Ly6G, CD11b, Siglec F, CD11c, Ly6C, CD24, CD103 and MHC class II. Data were acquired using a flow cytometer and analyzed by FlowJo software. Representative dot plots from a single mouse are shown. After excluding doublets and dead cells (**A**), dot plots were gated on CD45+ white blood cells (**B**). Neutrophils were identified based on their expression of Ly6G and CD11b (**C**). Non-neutrophils, Siglec F+ cells (**D**) were depicted on CD11b versus CD11c plots to identify eosinophils and alveolar macrophages (AM) (**E**). Ly6C+ monocytes were defined as non-neutrophils, Siglec F-CD11c-CD11b+Ly6C+ cells (**G**). Conventional DCs were identified as non-neutrophils, Siglec FCD11c+ CD24+ cells (**H**), and further classified based on their expression of CD103 or CD11b (**I**). CD24- cells within the CD11c+ gate (**H**) were then depicted on MHC class II versus autofluorescence (detected in the FITC channel) plots to identify exudate macrophages (ExM) and monocyte-derived DCs (mDC) (**J**).

**Figure S8.** Representative histograms displaying forward and side scatter expression on lung macrophages.  AM (left panels) and ExM (right panels) in the lungs of the indicated cohorts of mice at protocol D10 were assessed by flow cytometric analysis for relative size (assessed by mean fluorescent intensity of forward scatter; top panels),  and cell granularity (assessed by mean fluorescent intensity of side scatter; bottom panels).

**Figure S9.** Representative density plots and histograms displaying arginase and CD206 expression on lung macrophages.  AM and ExM in the lungs of the indicated cohorts of mice at protocol D10 were assessed for expression of arginase (upper density plots) and CD206 (lower histograms).

.


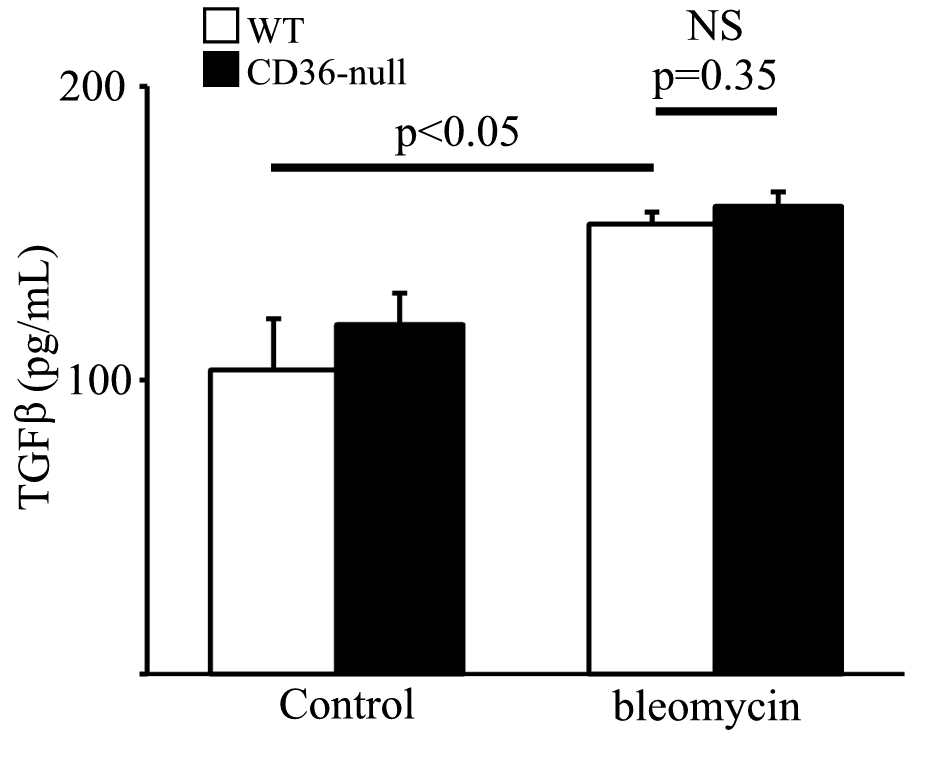


**Figure S10.** Twenty-four hours after WT and CD36-null bone marrow derived macrophages were treated with bleomycin (5 mU/ml) conditioned media was analyzed for TGFβ by ELISA. N=6, *p<0.05.


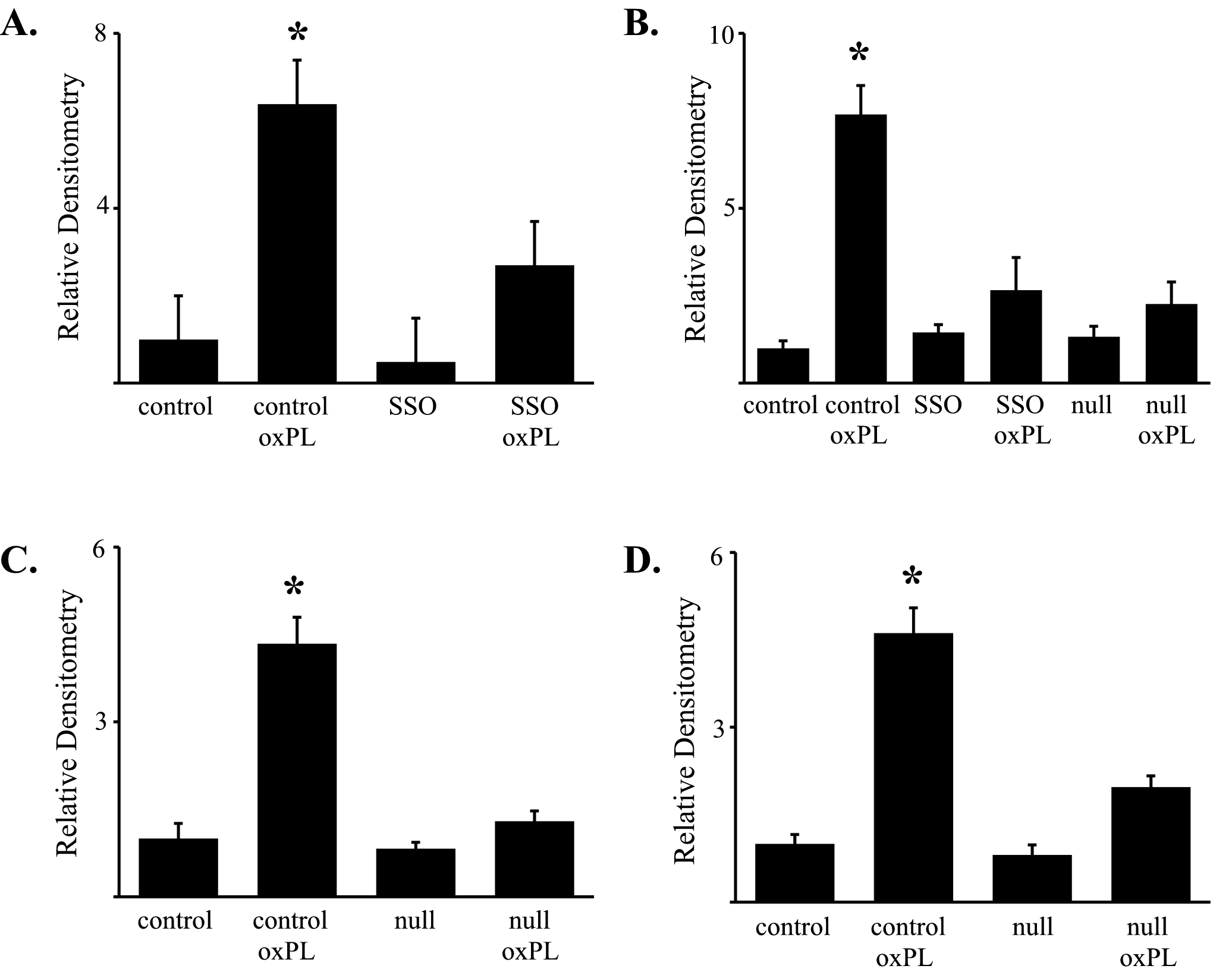


**Figure S11.** Relative densitometry for immunoblots in Figure 7. **A.** Level of Lyn from CD36 IP in Figure 7A. **B.** Level of p-Lyn in Figure 7B. **C.** Level of p-Lyn in Figure 7D. **D.** Level of p-Lyn in Figure 7E. N=4, *p<0.05 compare to other groups.


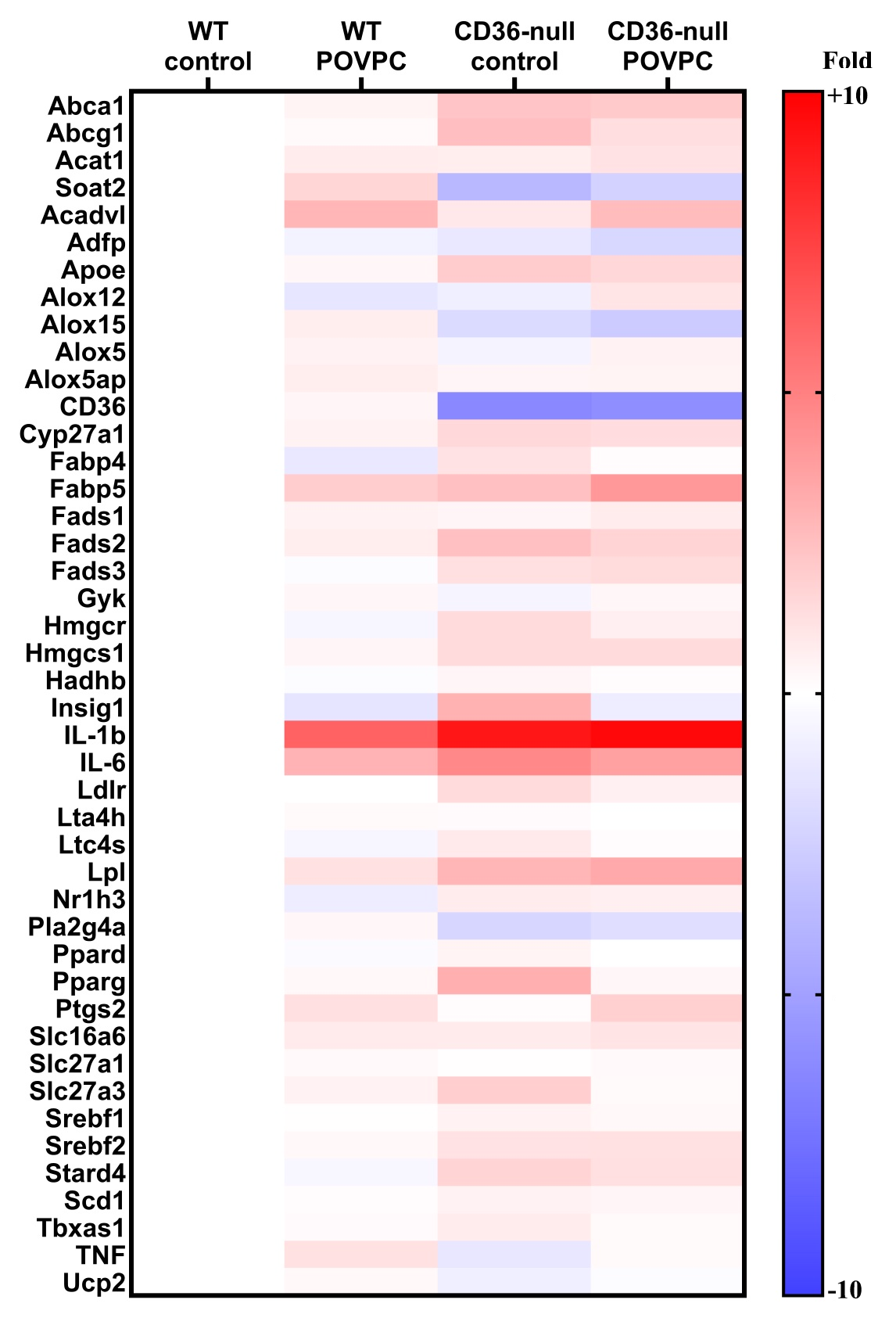


**Figure S12.** Relative gene expression of lipid regulated genes by bone marrow derived macrophages from WT and CD36-null mice treated with or without POVPC (10µg/mL). Values are expressed relative to WT control cells. N=4 per group.
